# Specificity of tilt illusion reduction through perceptual learning

**DOI:** 10.3389/fpsyg.2024.1346196

**Published:** 2024-03-18

**Authors:** Na-Ri Jeong, Seungmin Han, Hoon Choi

**Affiliations:** ^1^Department of Psychology, Hallym University, Chuncheon-si, Republic of Korea; ^2^Graduate School of Digital Contents, Hallym University, Chuncheon-si, Republic of Korea

**Keywords:** perceptual learning, tilt illusion, orientation, specificity, sports

## Abstract

Human perceptual ability can be improved by perceptual learning through repeated exposure or training. Perceptual learning studies have focused on achieving accurate perception of stimuli by improving perceptual sensitivity. However, eliminating illusions can also be one of the ways of accurate perception. To determine whether the illusion can be attenuated by perceptual learning, the current study used a tilt illusion where the orientation of the grating presented in the center (central grating) was misperceived because of the orientation of the grating presented in the periphery (surrounding grating). In Experiment 1, participants were trained either in the illusion training condition, in which they trained with illusory stimuli presenting both surrounding and central gratings together, or in the control training condition, where only the central grating was presented. The results confirmed that the tilt illusion was reduced only in the illusion training condition. Experiment 2 tested the transfer effect of learning, which is not often observed in perceptual learning. During training, the orientation of the surrounding grating was fixed to see whether the elimination of the illusion also occurred in the surrounding grating with an orientation that was not used during training. A decrease in the illusion was found only in the case of a surrounding grating with trained orientations, and not in the case of surrounding gratings with untrained orientations. These results suggest that the reduction in tilt illusion through training is due to perceptual learning.

## 1 Introduction

Although the goal of the human visual system is to identify and interpret an environment filled with a large amount of visual information in a short period of time, its ability is limited in many aspects, such that not all information can be fully processed. Perceptual learning, which can be defined as the long-term improvement of performance on perceptual tasks through repeated exposure or training, has been suggested as an effective way to overcome the limitations of the perceptual system (Karni and Sagi, 1991; Watanabe et al., 2001).

Perceptual learning studies have focused on whether perceptual learning can improve perceptual sensitivity (see Sasaki et al., 2010). Despite the ongoing debates, researchers have been repeatedly reported that observers can perceive visual stimuli with greater sensitivity after perceptual learning, which means perceptual learning allows observers to see the visual environment more accurately.

Is heightened perceptual sensitivity the only way to see more accurately? Sometimes we see a visual stimulus differently than it is in reality, even though the stimulus is suprathreshold enough to be perceived obviously. This is called an illusion. If perceptual learning helps us perceive more accurately, we should see the visual environment without the influence of illusions as a result of perceptual learning.

Studies have found that perceptual learning can reduce several illusions, such as the Muller-Lyer illusion (Rudel and Teuber, 1963), the size-weight illusion (Flanagan et al., 2008; Ernst, 2009) and the curveball illusion (Lee and Choi, 2021). However, it is still unclear whether the reduction in illusion is solely due to perceptual learning. A number of studies have focused on the role of expectation rather than improved perception in the reduction of illusion after repeated training. Research has shown that practice induces even the inverted size-weight illusion in which the bigger of two equally weighted objects was perceived as heavier (Flanagan et al., 2008). In particular, the feedback provided during training sessions not only improved perceptual abilities, but also encouraged the use of active strategies allowing participants to change their responses based on feedback, regardless of their actual perception.

In the current study, we investigated the role of perceptual learning in reducing illusions through repetitive training. In particular, we focused on the specificity of perceptual learning. The specificity of perceptual learning, where the learning effect occurs only on the trained stimulus, is a defining feature of perceptual learning. We conducted two experiments. In Experiment 1, we confirmed that an illusion can be reduced by perceptual learning using the tilt illusion, in which the orientation of a central grating is distorted by the orientation of the surrounding grating. In Experiment 2, we tested its specificity, that is, whether this reduction of the tilt illusion is specific to the trained orientation of the surrounding grating.

## 2 Experiment 1

To see whether the tilt illusion could be reduced by perceptual learning, we measured the magnitude of the tilt illusion effect before (i.e., pre-test) and after (i.e., post-test) training sessions, during which half of the participants repeatedly experienced the tilt illusion stimulus (i.e., illusion training group) whereas the other half of the participants did not (i.e., control training group). If the tilt illusion is reduced by perceptual learning training, then the learning effect will occur only in the illusion training group.

### 2.1 Methods

#### 2.1.1 Participants

Twenty undergraduate and graduate students at Hallym University participated in the experiment in exchange for monetary compensation. All the participants had normal or corrected-to-normal visual acuity, and reported no history of color perception problems. All subjects were naïve to the purpose of the experiment and signed an informed consent form approved by the Institutional Review Board of Hallym University.

#### 2.1.2 Apparatus

The experiment was conducted using the Psychophysics Toolbox (Brainard, 1997; Pelli, 1997) for Matlab (MathWorks, Natick, MA, United States) on a PC with an Intel(R) Core^TM^ i7-4790 3.60GHz CPU and a GeForce GTX 770 graphics card. All displays were presented on a 24-inch LED monitor (BenQ XL2420Z) with a resolution of 1,920 pixels × 1,080 pixels and a refresh rate of 144 Hz. Participants were positioned approximately 60 cm apart from the monitor so that the display subtended a visual angle of 48° by 28°. A chin rest was used to maintain the participants’ head position. The experiment was conducted in a darkened room.

#### 2.1.3 Stimulus

**Figure 1:**
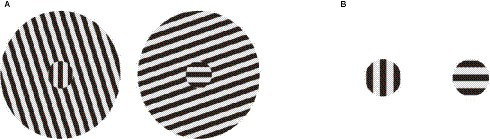
Two types of stimuli. **(A)** Illusion stimulus, comprised of a central grating and a surrounding grating, induced a tilt illusion. **(B)** Control stimulus had only a central grating.

Two types of stimuli were used, one of which induced the tilt illusion (i.e., illusion stimulus) and the other of which did not (i.e., control stimulus). The illusion stimulus consisted of the central grating subtending 1 × 1 degree of visual angle and the surrounding grating subtending 5 × 5 degrees (Figure 1A). The control stimulus consisted of the central grating only (Figure 1B). In both types of stimuli, the central grating was tilted in one of the following degrees: 0°, 3°, 357°, 87°, 90°, or 93°. In the illusion stimulus, the orientation of the surrounding grating was 162 ° (when the central grating orientation was 0°, 3°, or 357°) or 72° (when the orientation of the central grating was 87°, 90°, or 93°). All gratings had seven cycles per degree. All parameters of the stimuli were obtained from preliminary studies.

#### 2.1.4 Procedure

**Figure 2:**
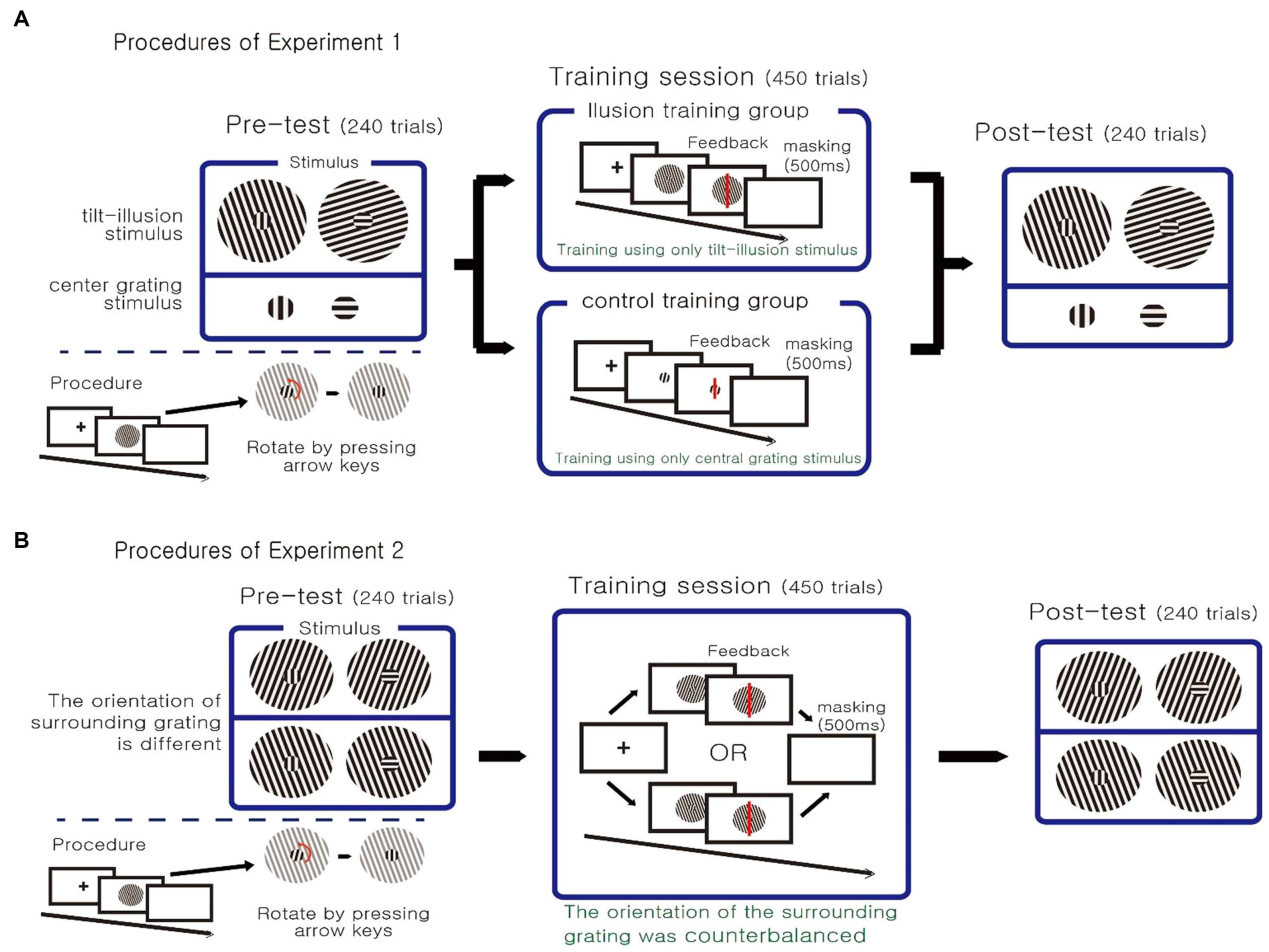
**(A)** Procedure of Experiment 1. The experiment consisted of seven sessions: a pretest, five training sessions and a posttest. Based on the type of the stimulus employed in the training sessions, participants were divided into two groups. **(B)** Procedure of Experiment 2. During training sessions, the orientation of the surrounding grating was fixed.

The experiment consisted of seven sessions: a pre-test, five training sessions, and a post-test (Figure 2). Participants were encouraged to attend the experiment at the same time each day, if possible, and were not allowed to be absent for more than two consecutive days. The participant’s task was to rotate the central grating of the presented stimulus until it was perceived as perfectly vertical or horizontal. At the beginning of the trial, the stimulus was presented in the center of the screen and the participant rotated the orientation of the central grating using the arrow keys on the keyboard. When the participants perceived that the central grating was perfectly vertical or horizontal, they ended the trial by pressing the space bar. At the end of the trial, white noise was presented as a masking stimulus. Participants controlled the progress of the experiment themselves. Participants could start a new trial on their own by pressing the space bar, and take sufficient rest time any time they wanted in addition to the programmed break time.

##### 2.1.4.1 Pre-test and post-test

On the first day (pre-test) and the last day (post-test) of the experiment, we measured the degree of illusion, which was defined as the difference between the reported orientation (i.e., the orientation of the central grating reported by participants as being vertical or horizontal) and the actual vertical or horizontal orientation. Both illusion and control stimuli were used. The experiment consisted of a total of 240 trials, each with 60 trials per block (30 trials of the illusion stimulus, and 30 trials of the control stimulus). The order of all trials within a block was completely randomized.

##### 2.1.4.2 Training session

From the second to the sixth day of the experiment, training sessions were conducted for a total of 5 days. The tasks and stimuli were identical to the pre- and post-test. However, in the training session, response feedback was given on each trial. Participants were randomly divided into two groups. The illusion training group was trained only with the illusion stimulus that induced the tilt illusion. In the control training group, only the control stimulus was used, in which only the central grating was presented without the surrounding grating. The experiment consisted of 90 trials per block, for a total of 450 trials.

### 2.2 Results

**Figure 3:**
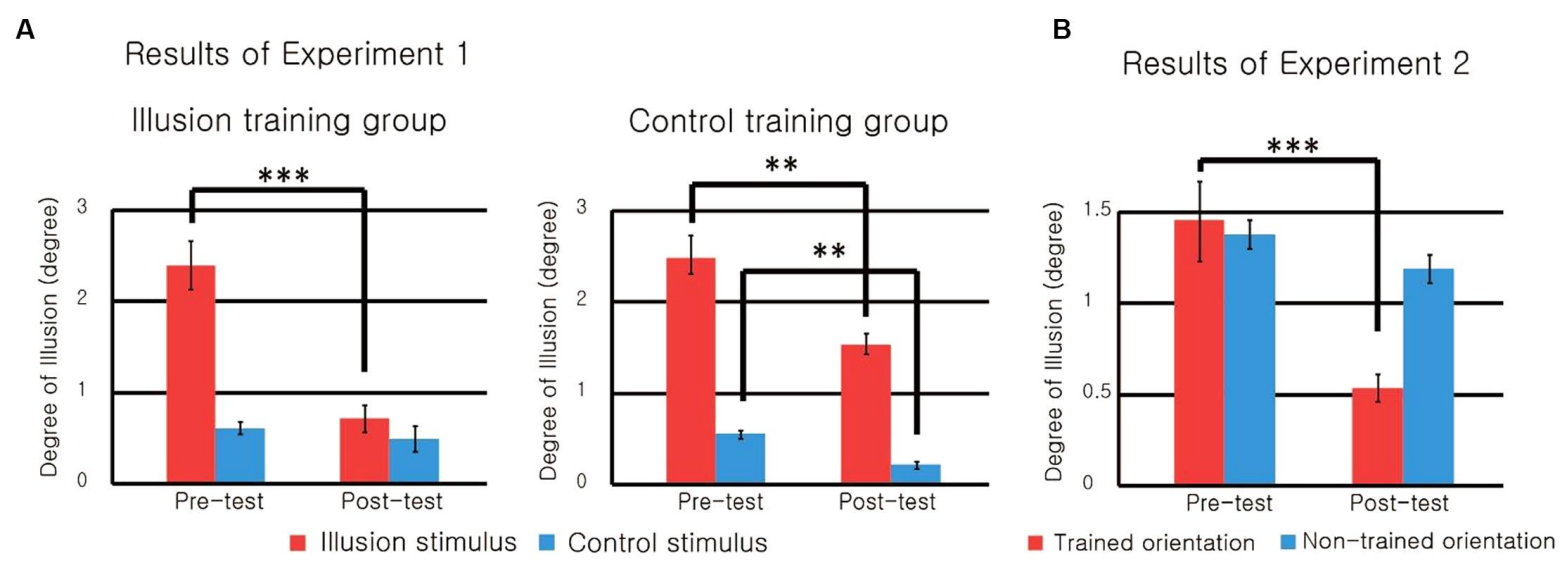
**(A)** Results of Experiment 1. In the posttest of the illusion training group, the illusion was reduced. **(B)** Results of Experiment 2. The reduction of the illusion was found only for the trained orientation (***p < 0.001, **p < 0.01).

Figure 3 shows the degree of tilt illusion for each condition. A three-way mixed model ANOVA revealed significant effects on all main factors: (a) type of stimulus (illusion stimulus, control stimulus), *F*(1, 18) = 108.840, *p* < 0.001, *η_p_*^2^ = 0.858, (b) training group (illusion training group, control training group), *F*(1, 18) = 279.035, *p* < 0.001, *η*_p_^2^ = 0.939, and (c) training(pre-test, post-test), *F*(1, 18) = 43.729, *p* < 0.001, *η_p_*^2^ = 0.708.

The ANOVA also showed significant two-way interactions between type of stimulus and the training group, *F*(1, 18) = 6.773, *p* = 0.018, *η_p_*^2^ = 0.273, and between type of stimulus and training, *F*(1, 18) = 62.563, *p* < 0.001, *η*_p_^2^ = 0.777, although the interaction between training group and training was not significant, *F*(1, 18) = 1.550, *p* = 0.229, *η*_p_^2^ = 0.079. In particular, the three-way interaction was significant, *F*(1, 18) = 19.471, *p* < 0.001, *η_p_*^2^ = 0.520.

The significant three-way interaction suggests differences in the training effect between the training groups. In the illusion training group, the degree of illusion for the illusion stimulus decreased significantly after the training (*p* < 0.001), but there was no significant difference for the control stimulus (*p* = 0.901). Meanwhile, in the control training group, where only the control stimulus was used during the training sessions, there was a significant decrease in the degree of illusion for both the illusion stimulus (*p* = 0.004) and the control stimulus (*p* = 0.001).

### 2.3 Discussion

This result suggests that different types of learning effects occurred depending on the stimulus trained. In the illusion training group, the degree of illusion decreased only for the illusion stimulus, and there was no decrease for the control stimulus, which was not used for training. In the pre-test, the degree of illusion for the control stimulus was low, so the lack of training effect for the control stimulus could be interpreted as a ceiling effect. However, the results of the control training group, which also significantly reduced the degree of illusion for control stimuli, are not consistent with this interpretation.

In the control training group, learning effects occurred for both types of stimuli. However, the learning effect for the illusion stimulus seems to have been a side effect of the learning for the control stimulus because training with the control stimulus could improve the performance of the orientation perception of the central grating. In fact, the learning effect for the illusion stimuli was less than that of the illusion training group.

The finding in this study that the tilt illusion was reduced as a result of training with repeated exposure to the illusion stimulus is consistent with previous studies showing that illusions can be reduced as a result of perceptual learning (Rudel and Teuber, 1963; Brosvic et al., 1997). However, it was still not clear whether the reduction of the tilt illusion was solely due to perceptual learning because we could not rule out the expectation hypothesis. Participants could easily distinguish the illusion stimulus with the surrounding grating from the control stimulus without the surrounding grating, and they had sufficient time to learn how to respond to the illusion stimulus during training sessions in which feedback was provided. In Experiment 2, we investigated whether specificity, one of the most important characteristics of perceptual learning, would be found in the reduction of the tilt illusion.

## 3 Experiment 2

One of the most distinctive characteristics of perceptual learning is the specificity of the learning effect. Improving perceptual abilities through perceptual learning is highly specific to the trained features, such as retinotopic location and orientation (Fiorentini and Berardi, 1980). In Experiment 2, we investigated whether the reduction of the tilt illusion in Experiment 1 was due to perceptual learning using specificity.

The result of Experiment 1, which showed that the tilt illusion was reduced by training could have been achieved in two ways. Either participants perceived the orientation of the central grating more accurately, or participants suppressed interference from the surrounding grating. The latter seems to be more appropriate, because no learning effect occurred for the control stimulus in the illusion training group.

In Experiment 2, we attempted to determine whether the suppression of the surrounding grating was specific to the trained orientation of the surrounding grating. After training the participants with a surrounding grating in a specific orientation, we checked whether the reduction of the illusion was also found even when a surrounding grating in an untrained orientation was presented. If the reduction of the tilt illusion was due to perceptual learning, the learning effect would occur only in the trained orientation.

### 3.1 Methods

#### 3.1.1 Participants

Fourteen undergraduate and graduate students at Hallym University who did not participate in Experiment 1 took part in the experiment.

#### 3.1.2 Apparatus and stimulus

Experiment 2 used the same apparatus and stimuli as Experiment 1 with the following exceptions. All stimuli used in the experiment were the illusion stimulus that induced the tilt illusion. The central grating had one of six orientations (0°, 3°, 357°, 87°, 90°, or 93°) as in Experiment 1, and the surrounding grating was presented with an orientation of 18° or 162°.

#### 3.1.3 Procedure

Experiment 2 used the same procedure as Experiment 1, but differed in the following respects. In the pre- and post-test, the surrounding grating was presented with an orientation of 18° or 162°, but had only one orientation in the training session. The orientation of the surrounding grating exposed during the training session was counterbalanced for each participant.

### 3.2 Results and discussion

Figure 3 shows the degree of illusion for each condition. A two-way repeated measures ANOVA revealed a significant main effect of training (pretest, posttest), *F*(1, 13) = 26.457, *p* < 0.001, partial *η*^2^ = 0.671, but no significant main effect of exposure (trained orientation, untrained orientation), *F*(1, 13) = 3.042, *p* = 0.105, partial *η*^2^ = 0.190. Interaction was also significant, *F*(1, 13) = 4.766, *p* = 0.048, partial *η*^2^ = 0.268. Significant interaction showed that the reduction in the tilt illusion had specificity, which means that the learning effect occurred only when the surrounding grating of the illusion stimulus had the trained orientation. The degree of the illusion for the trained orientation was significantly reduced after training (*p* = 0.001), but this was not the case for the untrained orientation (*p* = 0.366). This specificity supports the conclusion that the learning effect in this study was due to perceptual learning.

## 4 General discussion

In this study, we attempted to confirm the effect of perceptual learning on illusions through two experiments. In Experiment 1, we tested whether the illusion effect could be attenuated by training. The result showed that the tilt illusion was attenuated by training with repeated exposure to a tilt illusion stimulus. Experiment 2 confirmed whether the attenuation of the tilt illusion was solely due to perceptual learning. After training with the orientation of the surrounding grating fixed, we checked whether the training effect also occurred in the untrained orientation. As expected, we found a reduction in the illusion only for the trained orientation, with no significant effect for the untrained stimuli. This result shows that the reduction of the tilt illusion with training has specificity, a key feature of perceptual learning.

There has been controversy over whether performance improvement through repeated exposure training is purely due to improvements in perceptual abilities or is the result of involvement of relatively higher-level cognitive processes such as decision-making (see Watanabe and Sasaki, 2015). The main evidence cited by those who argue that perceptual learning is associated with improved perceptual ability is the specificity of perceptual learning, because specificity is a characteristic found early in the brain’s processing of visual information. In this experiment, we used this specificity to determine whether the reduction in the tilt illusion was due to an improvement in perceptual ability.

In particular, the reduction of contextual illusions, such as the tilt illusion, seems to result from attentional rather than perceptual learning. In the case of the tilt illusion, ignoring the influence of the surrounding grating is necessary to reduce the illusion, and research has shown that suppressing attention to task-irrelevant features can lead to learning to ignore them (Dixon et al., 2009). However, Experiment 2 demonstrates the specificity of the learning effect, suggesting that the reduction in contextual illusions may be due to perceptual learning, because the effects of perceptual learning are specific to stimuli that have been exposed or trained (Crist et al., 1997), whereas the effects of attentional learning can be generalized to untrained stimuli.

Unlike studies (e.g. Baccini et al., 2014) that used forced-choice tasks, this study used a task in which the orientation of the central grating was rotated until it was perceived as vertical or horizontal. Although our method has the advantage of providing a more accurate measure of the degree of the illusion, because the orientation of the surrounding grating was limited and feedback was provided, participants may have used some sort of rule-finding strategy to arrive at the correct answer. However, this seems unlikely given the results of Experiment 2, which showed a selective learning effect only for the orientation of the surrounding grating used in training.

Even when the same illusion stimulus was used in the pre-test of Experiments 1 and 2, the degree of illusion varied. This is due to the different orientations of the surrounding gratings used in Experiments 1 and 2. In Experiment 2, only two orientations were used, similar to the vertical orientation, which had less influence on the perception of the central grating with horizontal orientation, resulting in a smaller illusion in Experiment 2.

The results of this study that contextual illusions are weakened through training have great practical significance. In sports, contextual illusions can act as obstacles to sports performance. In the case of golf, the surrounding environment can affect the misperception of the degree of inclination of the place where the ball is placed. If optical illusions are weakened through training, more accurate judgments may benefit sports performance.

The finding that contextual illusions, such as the tilt illusion, are attenuated by training has important practical implications. In many sports, contextual illusions can act as a barrier to athletic performance. In golf, for example, the surrounding environment can induce a misperception of the slope of where the ball is placed. Attenuating the illusion through training could help athletes make more accurate judgments, which could benefit their performance.

## Data availability statement

The raw data supporting the conclusions of this article will be made available by the authors, without undue reservation.

## Ethics statement

The studies involving humans were approved by the Institutional Review Board at Hallym University. The studies were conducted in accordance with the local legislation and institutional requirements. The participants provided their written informed consent to participate in this study.

## Author contributions

N-RJ: Formal analysis, Investigation, Methodology, Software, Writing – original draft. SH: Data curation, Formal analysis, Visualization, Writing – review & editing. HC: Conceptualization, Funding acquisition, Methodology, Project administration, Supervision, Writing – review & editing.
